# Revamping Hepatocellular Carcinoma Immunotherapy: The Advent of Microbial Neoantigen Vaccines

**DOI:** 10.3390/vaccines12080930

**Published:** 2024-08-21

**Authors:** Junze Liang, Yanxia Liao, Zhiwei Tu, Jinping Liu

**Affiliations:** State Key Laboratory of Oncology in South China, Guangdong Provincial Clinical Research Center for Cancer, Sun Yat-sen University Cancer Center, Guangzhou 510060, China; liangjz1@sysucc.org.cn (J.L.); liaoyx2@sysucc.org.cn (Y.L.); tuzw@sysucc.org.cn (Z.T.)

**Keywords:** hepatocellular carcinoma, immunotherapy, microbial neoantigens, microbiome, tumor vaccines

## Abstract

Immunotherapy has revolutionized the treatment paradigm for hepatocellular carcinoma (HCC). However, its efficacy varies significantly with each patient’s genetic composition and the complex interactions with their microbiome, both of which are pivotal in shaping anti-tumor immunity. The emergence of microbial neoantigens, a novel class of tumor vaccines, heralds a transformative shift in HCC therapy. This review explores the untapped potential of microbial neoantigens as innovative tumor vaccines, poised to redefine current HCC treatment modalities. For instance, neoantigens derived from the microbiome have demonstrated the capacity to enhance anti-tumor immunity in colorectal cancer, suggesting similar applications in HCC. By harnessing these unique neoantigens, we propose a framework for a personalized immunotherapeutic response, aiming to deliver a more precise and potent treatment strategy for HCC. Leveraging these neoantigens could significantly advance personalized medicine, potentially revolutionizing patient outcomes in HCC therapy.

## 1. Introduction

According to the Cancer Statistics 2024, liver cancer is the sixth most common malignancy and has the third highest mortality rate worldwide, posing a significant global health challenge [1]. Hepatocellular carcinoma (HCC) constitutes the majority of liver cancer cases [2]. Despite recent advancements in systemic therapies for advanced HCC, the 5-year survival rate remains below 10%. Over the past 5 years, immune checkpoint inhibitors (ICIs) have revolutionized HCC management. However, due to low T-cell infiltration and a modest tumor mutational burden (TMB), HCC remains relatively immune-resistant [3]. In several clinical trials, ICIs have demonstrated response rates of approximately 12–18% as monotherapy [4,5,6,7]. Low tumor lymphocytic infiltration has been suggested as a mechanism underlying these poor responses to ICIs [8]. Consequently, there is an urgent need to develop strategies aimed at ‘heating’ these tumors and improve response rates to ICIs.

Neoantigens, conventionally derived from genetic mutations (such as single-nucleotide variants, insertions, and deletions), incomplete transcriptional splicing, alternative translations, or post-translational modifications, are presented on the surface of tumor cells by major histocompatibility complex (MHC) molecules. These neoantigens are recognized as neoepitopes by T cells, making them compelling targets for T cell-mediated immune responses. Personalized neoantigen vaccines for cancers show the potential to induce the infiltration of neoepitope-specific T cells into tumor tissues, thereby effectively targeting and eliminating cancer cells expressing these antigens [9]. To date, more than 100 clinical trials have validated the safety, feasibility and immunogenicity of personalized neoantigen vaccines in patients with both hypermutated or non-hypermutated cancers [10,11], including HCC [12]. Some phase III clinical trials are listed below(Table 1).

Microbial organisms play essential roles in various physiological processes within the human body and have been shown to influence the response to ICIs in patients with HCC [13,14]. Chronic infections with hepatitis B virus (HBV) or hepatitis C virus (HCV) are well-documented causes of HCC, contributing both directly and indirectly to immune-mediated carcinogenesis [15,16]. Preventative measures, such as HBV vaccination or antiviral treatments, have beneficial effects in reducing the incidence of HCC [17,18,19]. Recent studies have demonstrated that microbial elements can activate tumor-infiltrating lymphocytes by reprograming the tumor microenvironment or presenting bacterial-specific peptides [20,21]. Consequently, the identification of microbial markers predictive of survival benefits in HCC immunotherapy and the exploration of novel therapeutic targets to modulate the immunotherapy response represent pivotal areas of future research.

In this perspective, we aim to outline and propose a personalized immunotherapeutic approach that offers a more precise and potent treatment strategy for HCC. By harnessing the potential of these unique neoantigens, we envision significant advancements in the field of personalized medicine, with the potential to revolutionize patient outcomes in HCC therapy.

## 2. Tumor Neoantigen and Hepatocellular Carcinoma Immunotherapy

### 2.1. Neoantigens: Promising Targets for Cancer Therapy

Neoantigens, which are novel antigens produced by tumor cells due to various tumor-specific changes, represent a promising direction in cancer treatment [22]. These neoantigens can be generated through four main mechanisms: (i) genetic mutations, such as chromosomal translocations, insertions and deletions, and single-nucleotide variants in somatic cells; (ii) transcriptional activities, including alternative splicing; (iii) translational mechanisms, like ribosomal frameshifting and initiation from non-AUG start codons; and (iv) post-translational modifications, including antigen processing and proteasomal peptide splicing [23]. This study summarizes the common neoantigens, and a list of these neoantigens is presented below (Table 2).

These neoantigens, released from damaged or dying cancer cells, are subsequently taken up by professional antigen-presenting cells (APCs). In the lymph nodes, APCs cross-present these cancer-associated antigens via MHC class I molecules, leading to the priming of CD8^+^ T cells in a process known as cross-priming. Effector CD8^+^ T cells then migrate to the tumor site, where they exert their cytotoxic effects in an antigen-dependent manner, potentially enhancing immunity through epitope spreading. CD4^+^ T cells, primed by MHC class II presentation on APCs, can assist CD8^+^ T cells, induce MHC class I expression on cancer cells, activate myeloid cell cytotoxicity, and directly kill tumor cells [24,25]. For cancer vaccines, it is essential to consider all possible sources of neoantigens.

The tumor mutational burden (TMB) is often associated with the number of neoantigens [26]. Compared to heavily mutated tumors such as melanoma or lung cancer, hepatocellular carcinoma (HCC) shows a low to intermediate TMB (five mutations per megabase) [27]. Commonly altered pathways in HCC include telomere maintenance (~60%), Wnt/β-catenin (~54%), PI3K/AKT/mTOR (~51%), TP53 cell cycle (~49%), Ras/MAPK (~43%), epigenetic regulation (~32%), chromatin remodeling (~28%), and oxidative stress (~12%) [28]. Using a combination of whole exome sequencing, RNA-seq, and computational bioinformatics, Yang et al. suggested that TP53-specific neoantigens are linked with overall survival in HCC patients [29]. Another study examined 363 HCC cases using whole-exome sequencing and DNA copy number analyses, along with 196 HCC cases through DNA methylation, RNA, miRNA, and proteomic profiling. This study identified significantly mutated genes, such as LZTR1, EEF1A1, SF3B1, and SMARCA4, and developed a p53 target gene expression signature that correlates with poor survival [30]. Identifying potential neoantigens and understanding their mechanisms are crucial for developing therapeutic targets and enhancing HCC clinical management.

### 2.2. Immune Checkpoint Inhibitors for Hepatocellular Carcinoma Immunotherapy

Over the past decade, immune checkpoint inhibitors (ICIs) have ushered in a new era in the treatment of HCC [2,31]. These ICIs modulate anti-tumor T-cell responses and are expressed not only on T cells but also on antigen-presenting cells (APCs) such as dendritic cells (DCs) and macrophages, as well as on tumor cells. Critical immune checkpoints, like PD-1, PD-L1, CTLA4, LAG3, and TIM3, play vital roles in limiting T-cell activity and maintaining self-tolerance [32,33,34]. The primary mechanism involves the interaction between PD-1 and PD-L1, leading to the dephosphorylation of T cell-activating kinases, which results in T-cell inactivation [32,35]. Blocking PD-1 and PD-L1 restores the function of effector CD8^+^ T cells [36]. Anti-CTLA4 works at the T cell–APC immune synapse by promoting the unopposed interaction of B7 costimulatory ligands with CD28, thereby enhancing the activation of CD4^+^ and CD8^+^ T cells and rebalancing the regulatory compartments within the tumor microenvironment (TME) [37]. LAG3 inhibits effector T-cell activity via the KIEELE motif, which is functionally associated with Treg cell-mediated immunosuppression [38]. TIM3, by binding to galectin 9 or CEACAM1, induces CD8^+^ T-cell apoptosis and exhaustion [39].

Despite significant improvements in survival outcomes with ICIs, the majority of HCC patients do not experience lasting benefits from these treatments. For example, the phase III clinical trial CheckMate 459 demonstrated that nivolumab (anti-PD1) monotherapy did not significantly extend overall survival compared to sorafenib in patients with advanced HCC, with an objective response rate (ORR) of only 15% [4]. Similarly, in the KEYNOTE-224 clinical trial, pembrolizumab (anti-PD1) monotherapy exhibited an overall ORR of just 17% [7]. Anti-PD-L1 monotherapies, such as atezolizumab and durvalumab, showed ORRs of 17% and 11%, respectively, in HCC patients [40]. The phase I/II study confirmed that tremelimumab (anti-CTLA4) had an ORR of 9.5% in patients with unresectable HCC [41]. Several ICI combinations have progressed to phase III clinical trials, where they are being compared to the standard of care in patients naive to systemic therapy. For instance, the CheckMate 040 trial reported an ORR of 32% for nivolumab plus ipilimumab in patients with advanced HCC [42]. Atezolizumab combined with bevacizumab [43] and tremelimumab plus durvalumab [44] have now been established as first-line therapies in this context.

In conclusion, single-agent ICIs have demonstrated an ORR of 15–20% in patients with advanced HCC, often without a significant overall survival benefit [45]. Generally, ORRs range from 15 to 30% with monotherapies to more effective combination therapies in HCC patients. Additionally, approximately 30% of HCC cases exhibit intrinsic resistance to ICIs, potentially due to antigen-loss variants and low TMB, resulting in fewer neoantigens, which make them less recognizable to the immune system [46].

### 2.3. Neoantigen Vaccines in Hepatocellular Carcinoma

Neoantigen vaccines represent a promising immunotherapeutic strategy designed to bolster the efficacy of ICIs in HCC patients exhibiting low levels of tumor-infiltrating lymphocytes. Resident memory CD8^+^ T cells are important for tumor control and can be replenished by circulating CD8^+^ T cells [47,48]. However, neoantigen-specific circulating CD8^+^ T cells are detected in only approximately 15% of HCC patients [49]. To address this, various neoantigen vaccine platforms have been developed and tested in clinical trials, including peptide-based, RNA-based, DNA-based, and dendritic cell (DC)-based vaccines combined with adjuvants. At the time of this review, a search of a clinical trial (4 July 2024, http://clinicaltrials.gov/) database using the terms “cancer” and “neoantigen vaccine” yielded 10 completed clinical trials, 16 active but not recruiting trials, and 54 currently recruiting clinical trials.

From this retrieval, this review collates the findings from several clinical trials investigating neoantigen vaccines in HCC, highlighting their development and therapeutic potential.

### 2.4. The Relevance between Personalized Neoantigen Vaccines and Immune Checkpoint Inhibitors Response

Recent studies have highlighted the synergistic potential of personalized neoantigen vaccines combined with ICIs. Chen et al. demonstrated that a personalized neoantigen vaccine, in conjunction with PD-1 blockade, significantly increased CD8^+^ tissue-resident memory T-cell infiltration in preclinical HCC models [50]. In a phase I/II trial (NCT04251117), the personalized DNA vaccine GNOS-PV02, encoding up to 40 patient-specific neoantigens, was administered alongside pembrolizumab and plasmid-encoded IL12, resulting in a 25% response rate in patients with advanced HCC [51]. Notably, neoantigen-specific T-cell responses were detected in 19 out of 22 patients (86.4%) using enzyme-linked immunosorbent spot assays (ELISPOT). Multiparametric cellular profiling further revealed that vaccine-specific CD4^+^ and CD8^+^ effector T cells were active, proliferative and cytolytic [52].

## 3. Microbial Impacts on Hepatocellular Carcinoma Neoantigen and Immunotherapy

### 3.1. Virus

A total of 1280 virus types have been identified in the blood of HCC patients, underscoring the strong association between viral infection and HCC [15,53]. Despite over two decades of universal vaccination, HBV and HCV remain the most significant global risk factors for HCC, particularly in Asian populations [54]. These neoantigens, potential targets for neoantigen vaccines in HCC, may arise during host–viral interactions, viral immune responses, and viral integration into the host genome [55]. However, the carcinogenic mechanisms of HBV and HCV differ. HBV can integrate into the host genome, while HCV induces HCC through chronic inflammation and liver cirrhosis, suggesting HBV-induced HCC have more neoantigens than HCV-induced HCC [56].

HBV, the non-cytopathic and double-stranded hepatotropic DNA virus, impacts host genomic instability [57]. Integration of the HBV genome causes host genomic instability and the formation of novel fusion transcripts, resulting in microdeletions, duplications, inversions or rearrangements of host genomic sequences. Numerous events of HBV genome integration into the host genome have been recorded in HCC cases, particularly near ribosomal protein genes, telomerase reverse transcriptase, mixed lineage leukemia family genes, calcium signaling genes and apoptosis-associated genes [58].

Among these genes, telomerase reverse transcriptase (Tert) promoter mutation, with an overall frequency of approximately 75%, is one of the most common microdeletions in HCC. There have been three cases of HBV DNA integrations around Tert, including 0.8 kb upstream to the promoter, 0.3 kb and 16 kb downstream of the gene [58]. As one of the earliest switches in the transformation of HCC, the mutation of Tert activates telomerase activity, allowing continuous cell division. In glioblastoma, Elisa Aquilanti found telomerase inhibition was an effective therapeutic strategy for treating Tert promoter-mutant types [59]. Though telomerase vaccines, such as GV1001, have shown inefficiency, vaccines that may induce Tert immunogenicity remain a hot research topic for HCC therapy [60]. 

Notably, researchers have advanced neoantigen vaccine technology by expressing neoantigens in chimeric HBV core antigens, which selectively induce tumor-specific CD8^+^ T-cell activation. The HBV core antigen serves as a versatile platform for expressing and carrying neoantigens due to its ability to form virus-like particles (VLPs) that are highly immunogenic. The foreign epitope sequence is engineered into a specific region of the HBV core antigen, typically the major immunodominant region (MIR), which is highly accessible to the immune system. These expressing particles mimic the structure of a virus, enhancing the presentation of the neoepitope to the immune system. This structure facilitates the activation of CD8^+^ T cells, which are crucial for targeting and destroying tumor cells [61].

In a study, a Db/Sp244-252/R251H (R mutated to H) neoantigen epitope was engineered in the EndoB2-Sp protein [62]. A single injection of this EndoB2-Sp expression vector into C57Bl/6j mice effectively activated IFN-γ^+^ CD8^+^ T cells targeting the neoantigen epitope [62]. This method, using the assembly-deficient HBV core antigen to express Db/Sp244-252/R251H, generated a substantial CD8^+^ T-cell response compared to the EndoB2-Sp vaccine alone [62].

Overall, the theoretical basis supports using neoantigens for immunotherapy in HBV-induced HCC. Compared to non-HBV-induced HCC, HBV-induced HCC is more likely to generate neoantigens due to genomic instability caused by HBV [63]. Additionally, the HBV core antigen can serve as an effective carrier for neoantigens, enhancing their immunogenicity.

### 3.2. Bacterial

The gut microbiota comprises approximately a trillion microorganisms, including bacteria, viruses and fungi, forming a highly diverse micro-ecosystem [64]. Among these, bacteria are the most abundant and diverse microorganisms, significantly influencing the gut–liver axis [65]. The regulation of gut microbiota affects HCC more profoundly than other cancers [66].

Leaky gut and dysbiosis increase intestinal permeability, allowing more microbial metabolites and microbiota-associated molecular patterns (MAMPs) from the gut to enter the liver [66]. Excessive microbial and MAMPs damage liver cells and induce inflammation, ultimately leading to liver cirrhosis and HCC [67]. One such MAMP, lipopolysaccharide (LPS), primarily originates from the outer membrane of Gram-negative bacteria. When leaky gut and dysbiosis occur, LPS enters the liver from the gut, activating Kupffer cells and prompting chronic inflammation. This process leads to liver cirrhosis, creating favorable conditions for HCC development [68,69].

Researchers have validated that specific gut microbiota communities can enhance the efficacy of ICIs [66]. In some tumors, antibiotic use negatively impacted the response to immune checkpoint blockade (ICB), evidenced by lower overall survival rates in patients treated with antibiotics during immunotherapy [66]. In addition, transplanting fecal microbiota from cancer patients who responded to ICB into germ-free mice improved the anti-tumor effects of immunotherapy, whereas fecal microbiota transplantation from non-responding patients failed [70]. However, these studies remain at the individual microbial level, and research into microbe–tumor interactions at the molecular level is equally valuable.

Seong-Young Kwon summarized four approaches of bacteria-mediated cancer immunotherapies (BMCIs) that induce tumor regression: enhancing the anticancer immune response, inducing apoptosis, disrupting cell metabolism and delivering therapeutic agents [71]. Researchers found that bacterial-derived peptides within tumor cells could activate tumor-specific T cells through antigen presentation due to the molecular mimicry of cancer cells by bacteria [71]. Peptide epitopes mimicking tumor-specific antigens were identified in *Enterococcus hirae* [72] and *Bifidobacterium breve* with specific prophage [73]. Additionally, peptides derived from the *Firmicutes* and *Bacteroidetes* showed homology to tumor-associated antigens in primary sequences, structure, and conformation, implying their potential as targets to assist ICIs [74]. However, the microbial mimicry activating anti-tumor immune response is not guaranteed. One factor is that bacterial-derived peptides may not be randomly structured, which reduces their diversity. The second factor is that the concentration of neoantigens may not be high enough to modulate anti-tumor immune response [75].

Beyond bacterial-derived peptides, MAMPs, which are immunostimulatory factors including cytokines or immune modulators, also have this characteristic [76]. For instance, *Vibrio vulnificus* flagellin *B* (FLAB) was used to activate immune cells such as macrophages and neutrophils against cancers via the TLR4 pathway [77].

**Figure 1:**
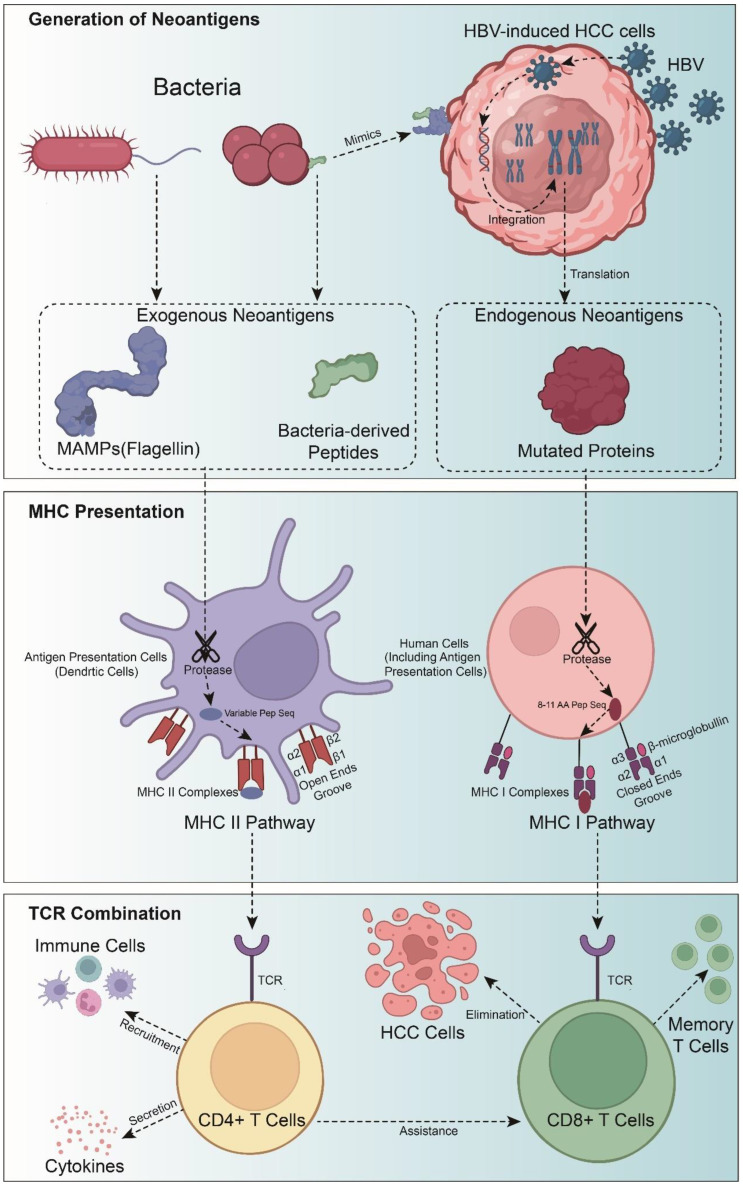
T-cells recognition neoantigens in HBV-induced hepatocellular carcinoma cells. Neoantigens generated by HBV DNA integration are presented by MHC Class I molecules, leading to the activation of CD8^+^ T cells. MAMPs and bacteria-derived peptides from bacteria activate CD4^+^ T cells through MHC Class II presentation.

Given that HCC patients often exhibit dysbiosis, it is speculated that dysbiosis might contribute to the failure of immunotherapy in some patients. Therefore, the modulation of gut microbiota may have a more profound impact on HCC than on other tumors, presenting an opportunity to develop novel antigen vaccines using bacteria derivatives to enhance the effectiveness of HCC immunotherapy (Figure 1). However, clinical trials of microbial neoantigen vaccines have not yet been reported to date.

## 4. Personal Neoantigen Cancer Vaccines Strategies for Hepatocellular Carcinoma: A Novel Approach

In response to the growing demand for personalized therapies, neoantigen cancer vaccines have demonstrated strong targeting capabilities, durable immune responses, and safety across various cancer types [78]. Establishing a standardized strategy for personal neoantigen vaccines in HCC immunotherapy is, therefore, highly significant. The essence of this strategy lies in enhancing vaccine quality based on the unique characteristics of individual HCC patients. Generally, the quality of these vaccines is associated with factors such as foreignness, clonal distribution, driver mutations, MHC presentation and T-cells receptor (TCR) avidity [79]. Additionally, given the polymorphism of immune systems among different patients, machine learning and AI present feasible methods to minimize resource and time wastage, thereby achieving personalized immunotherapy [80].

### 4.1. Discovery of Neoantigens

Currently, the discovery of neoantigens predominantly relies on next generation sequencing (NGS) technology. Researchers utilize NGS to sequence tumor and non-tumor tissues, identifying genes with non-synonymous mutations for neoantigen vaccine design [81]. The advent of single molecule real-time sequencing (SMRT) has facilitated the alternative splicing and mutation events, thereby uncovering additional tumor-specific antigens (TSAs) [56]. Moreover, single-cell sequencing technology can identify driver genes and characterize clonal distributions, both crucial for the successful construction of neoantigen vaccines [56].

However, the multistep process from gene transcription to protein translation often leads to significant discrepancies between the genome and proteome [82]. Consequently, leveraging proteomics to discover TSAs appears to be a direct and intuitive approach [83]. The rapidly advancing field of proteomics, enabled by high resolution LC-MS/MS technology, such as ASTRAL, is becoming increasingly vital for tumor neoantigen vaccine development [83,84]. The high coverage, sensitivity and resolution of ASTRAL allow the identification of lower abundant proteins, reduced sample loading amount, faster detection time and higher quality spectra. These attributes have enabled traditional genomic technologies to be applied within proteomics. Consequently, single-cell proteomics [85], spatial proteomics and peptide de novo sequencing technology are rapidly emerging and evolving.

In complex HCC tumor samples containing numerous exogenous microbial peptides and frequent mutations, peptide de novo sequencing assembles peptide sequences directly from spectra without being constrained by a reference database [86]. High-quality spectra with high resolution and good continuity of captured ions result in identified peptides with high confidence and integrity [87]. Commonly used peptide de novo sequencing applications include pNovo and PEAKS, which have been extensively validated and demonstrate advantages in analyzing complex samples [88,89].

### 4.2. Selection of Immunogenic Neoantigens

Not all neoantigens are suitable for vaccine development, making the selection of immunogenic neoantigens a critical strategic step. The criteria for selection can be summarized into five aspects: foreignness, clonal distribution, driver genes, MHC presentation, and TCR avidity [90]. The detailed introduction of the five aspects is as follows.

Foreignness refers to the degree of divergence between the neoantigen and its corresponding wild-type protein. The disparity means the possibility that the neoantigen will be recognized as non-self by the immune system. This heightened recognition enhances its potential as an immunogenic neoantigen.

Clonal mutations present in most cancer cells are preferred because they ensure that the immune therapy targets the entire tumor.

Driver mutations are genetic changes that promote cancer development by giving cells a growth advantage, making them more aggressive and better able to survive. These mutations are crucial for tumor survival, making them key targets for cancer therapies.

In contrast, passenger mutations are incidental changes that do not contribute to cancer growth or survival. They accumulate as the tumor evolves but are not involved in driving the cancer’s progression. While they can be recognized by the immune system, they are generally less important for therapeutic targeting. Hence, driver mutations are widely distributed in cancer cells.

**Figure 2:**
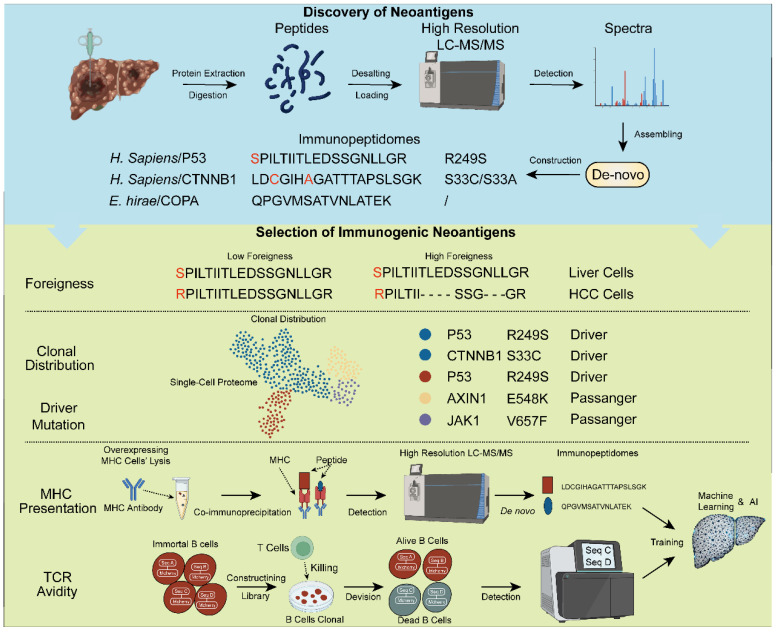
Neoantigens discovery and selection of immunogenic neoantigens. Utilizing proteomic techniques to identify neoantigens; developing training datasets for machine learning models to predict immunogenic neoantigens through multi-omics integration.

Homology is an effective method to assess foreignness of neoantigens while single-cell omics are both suitable for characterizing HCC clonal distribution and identifying driver genes (Figure 2) [79,90].

MHC presentation and TCR combination are the critical bottleneck in selecting immunogenic neoantigens. The primary role of MHC, categorized into Class I and Class II, is the antigens presentation to activate T cells [91].

The functions of MHC I and II are different. MHC class I primarily presents antigens originating from intracellular such as virus-infected cells or cancer cells, while MHC class II predominantly presents antigens from extracellular [79]. In addition, MHC class I ultimately presents endogenous to CD8^+^ T cells, whereas MHC class II presents exogenous antigens to CD4^+^ T cells [92] (Figure 1).

Experiments to screen neoantigens with high MHC affinity and TCR avidity have emerged; however, these methods consumed time and resources. Accelerating the selection of immunogenic neoantigens using computational technologies is also crucial for achieving personalized immunotherapy. These experimental results are applicable data for training machine learning models (Figure 2).

## 5. Ongoing Challenges in the Development of Neoantigen Vaccines for Hepatocellular Carcinoma

Tumor neoantigens are recognized as promising therapeutic targets for cancer immunotherapy due to their specific expression in tumor tissues. Personalized tumor vaccines derived from mutant neoantigens have demonstrated significant clinical efficacy in trials for glioblastoma, renal cell carcinoma, colorectal cancer, non-small cell lung cancer, and melanoma. Patients treated with neoantigen vaccines have exhibited vaccine-related tumor regression, suppressed tumor recurrence, and prolonged survival [93,94].

However, due to the heterogeneity of tumors and the polymorphism of the immune system, each patient generates the library of neoantigens, presenting a significant bottleneck in the clinical application of neoantigen vaccines. Personal neoantigen vaccines, requiring a process of development with high efficiency, offer an effective solution by tailoring the vaccine to each patient. In this study, it is proposed that accelerating the process by proteomics and computational technologies is essential for achieving personalized immunotherapy.

Nonetheless, the development and application of HCC neoantigen vaccines continue to encounter substantial challenges. The initial step in vaccine development involves identifying neoantigens through omics technologies. Although de novo peptide identification in HCC is feasible, it remains fraught with challenges. One such challenge is the difficulty in distinguishing between amino acid pairs with similar masses (e.g., glutamine (Q) and lysine (K)). For instance, if a de novo tool predicts Q but the actual amino acid is K, the mass difference is less than 0.05 Da, rendering the identification erroneous [95]. Accurate assembly of peptide sequences is crucial for neoantigen discovery, as any discrepancies in amino acids could result in ineffective vaccines. Post-translational modifications (PTM) add another layer of complexity, with more than 200 types identified, including phosphorylation and acetylation [96]. Although considering PTMs can potentially enhance the detailed resolution of de novo tools, it also increases computational complexity. As the algorithm needs to handle a complicated calculation and potential mismatches, this could affect the overall accuracy of identification in certain circumstances, especially when validation data are limited or the algorithm is not optimally tuned [97]. Although high-resolution LC-MS/MS data have improved, currently de novo tools do not fully utilize this precision, necessitating continuous updates to these tools [95].

Additionally, challenges in MHC presentation and TCR avidity impeded the development of neoantigen vaccines. The diversity of MHC and TCR impacts the universality of neoantigen vaccines [98,99]. Moreover, high MHC affinity of neoantigens does not necessarily equate immunogenicity, as TCR recognition is a complex, multistep process [100]. Screening experiments for neoantigens recognized by T cells are labor-intensive and resource-consuming. Therefore, while these experiments showed promise, they are not yet suitable for personalized HCC treatment, underscoring the importance of predictive tools. Current MHC affinity prediction tools have poor accuracy, with one meta-analysis showing that only 2.7% of predicted neoantigens were recognized by patient-derived T cells [101]. Similar outcomes were reported by Tumor Neoantigen Selection Alliance (TESLA) global consortium, where only 37 out of 638 predicted neoantigens were recognized by the patient T cells [102]. Thus, enhancing the accuracy of these prediction tools through more extensive training data is essential.

## 6. Conclusions

Hepatocellular carcinoma (HCC) ranks as one of the top six cancers globally with the third highest mortality. Despite the transformative impact of immune checkpoint inhibitors (ICIs) over the past five years, HCC remains an immune-resistant tumor, with ICIs showing low response rates as monotherapies in numerous clinical trials. Personal neoantigen vaccines for tumors can enhance specific T-cell infiltration, and when combined with immunosuppressants, they demonstrate superior efficacy in eradicating tumor cells compared to monotherapy [103]. Neoantigen vaccines have shown significant efficacy in treating various cancers, including pancreatic cancer, melanoma, non-small cell lung cancer, and glioma [104,105,106,107,108]. In the phase III clinical trial named NCT03897881, compared to pembrolizumab (anti-PD-1) monotherapy to melanoma, the combination of mRNA-4157 (V940) with pembrolizumab resulted in a longer recurrence-free survival, with a hazard ratio (HR) for recurrence or death of 0.561 [109]. Additionally, the 18-month recurrence-free survival rate was 79% in the combination group compared to 62% in the monotherapy group [109]. These findings suggest that neoantigen vaccines may also hold potential for treating HCC. However, research on personalized vaccines for HCC remains limited. This underscores the urgent need for strategies to ‘heat’ HCC to enhance responsiveness to ICIs. This review aims to provide insights into the development of tumor neoantigen vaccines for HCC by summarizing current research and clinical applications of these vaccines.

Neoantigens, newly formed antigens created by tumor cells in response to specific mutations, are taken up by antigen-presenting cells (APCs). Within lymph nodes, these APCs cross-present neoantigens via MHC class I, which activates CD8^+^ T cells. These effector T cells then travel to the tumor site, where they target and destroy cancer cells in an antigen-dependent process that boosts immunity through epitope spreading. Additionally, CD4^+^ T cells, activated by MHC class II on APCs, support CD8^+^ T cells, recruit immune cells and secrete cytokine to anti-tumor cells.

Though HCC is immune-resistant, it exhibits a strong relationship with microbial elements that may activate the immune system. Due to HBV genomic integration, HBV-induced HCC has numerous mutations, thus generating endogenous neoantigens. Moreover, HCC patients often suffer dysbiosis, leading to gut microbiota entering the liver via a portal vein. Studies have found that some bacteria can mimic peptides derived from tumor cell surfaces, thereby producing exogenous neoantigens. Therefore, while HCC currently presents as an immune-resistant tumor, the neoantigens it contains offer numerous possibilities for activating immunotherapy with neoantigen vaccines.

Given the heterogeneity of HCC and the variability of human immune system, personal neoantigen vaccines tailored to individual HCC patients appear more suitable. The discovery and immunogenic selection of neoantigens are essential to construct personal neoantigen vaccine strategies for HCC. Neoantigen discovery is typically focused on the genome, which may not be as direct as the proteome, as proteins are the direct executors of biological functions. High resolution LC-MS/MS, enriching proteomic technologies, provides a foundation for identifying neoantigens through more precise proteomic analysis. De novo peptide technology can directly assemble peptides from spectra without relying on reference databases, making it suitable for HCC neoantigen research due to its high microbial diversity and numerous mutations. However, the preparation of neoantigen vaccines requires accurate peptide sequences, necessitating the advancements in de novo tools to improve accuracy, such as distinguishing PTMs and amino acids with close masses. Furthermore, as LC-MS/MS resolution continues to increase, peptide de novo tools must more efficiently utilize high-resolution spectra.

MHC presentation and TCR avidity are key stages for screening out immunogenic neoantigens. Currently, experiments to screen out immunogenic neoantigens are too complex to meet personal demands. Although many immunogenic prediction tools have emerged, they primarily focus on predicting MHC class I affinity of neoantigens, and their accuracy remains questionable. Two factors contribute to their low accuracy: the construction of many prediction tools is based on the genome, leading to discrepancies between the genome and proteome that cause errors, and the confusion of TCR recognition and MHC affinity. Overcoming the aforementioned bottlenecks in screening immunogenic neoantigens may be possible by combining experiments to develop prediction tools for each step, specifically for forecasting TCR recognition.

## Figures and Tables

**Table 1:** Neoantigen vaccines in phase III clinical trials.

Clinical Trials ID	Disease	Vaccine Type	Enrollment
NCT01989572	Melanoma	Peptide	815
NCT00676507	Non-small cell lung cancer	Gene-modified cells	532
NCT00324831	Diffuse large B-cell lymphoma	Tumor cell derivative vaccine	480
NCT00425360	Pancreatic cancer	Peptide	1110

**Table 2:** Common neoantigens in HCC. The neoantigens are required to be identified at least in 10 HCC tissues of samples in the COSMIC database, and the type of mutation is non-synonymous mutation (* indicates a stop codon).

Protein	Mutation
ANK2	p.H724N
AXIN1	p.E464K, p.K219 *, p.W85 *
CNTN5	p.D657Y
CSMD1	p.G2864S, p.G2922S
CSMD2	p.R1076H
HERC1	p.A3233T
MUC4	p.T203M, p.T4235A
MYO10	p.E329A
OBSCN	p.F5062Y, p.R6669H, p.R7626H, p.E3824V, p.R2457L, p.F6019Y
PCLO	p.P585L, p.S676C
PKHD1	p.A1484S, p.E406G
PTPRB	p.T341I, p.N150K, p.T559I
PTPRQ	p.P1735S
ROBO2	p.R70 *
RYR1	p.H735Y
RYR3	p.G2045 *, p.H1519L, p.R3557P
SDK1	p.Q2189H, p.S1017P
SPEG	p.A68E
SYNE1	p.G1152C
TSC2	p.Q35 *, p.A100V, p.S809Lfs * 7, p.K14N, p.Q63 *, p.E450 *, p.E96 *

## Data Availability

The information of clinical trials was collected from https://clinicaltrials.gov/ and the discovered neoantigens were acquired from COSMIC database (https://cancer.sanger.ac.uk/cosmic/).
